# β-catenin-dependent High Bone Mass Induced by Loss of APC in Osteoblasts Does Not Require Lrp5 or Lrp6

**DOI:** 10.17912/micropub.biology.001000

**Published:** 2023-10-15

**Authors:** Cassandra R. Diegel, Megan N. Michalski, Bart O. Williams

**Affiliations:** 1 Department of Cell Biology, Van Andel Institute; 2 Department of Cell Biology and Director, Core Technologies and Services, Van Andel Institute

## Abstract

The requirement for LRP5 and LRP6 to prevent β-catenin degradation in the absence of the tumor suppressor APC is unclear because cell culture models have yielded conflicting results. We previously established that osteoblast-specific loss of APC causes β-catenin accumulation and increased bone mass, while loss of both LRP5 and LRP6 reduces bone mass. We report here that the simultaneous loss of APC, LRP5, and LRP6 in osteoblasts in mice phenocopies the APC osteoblast-specific knockout. Thus, β-catenin stabilization and increased bone mass after loss of APC in osteoblasts in vivo are not dependent on LRP5 and LRP6.

**Figure 1.**
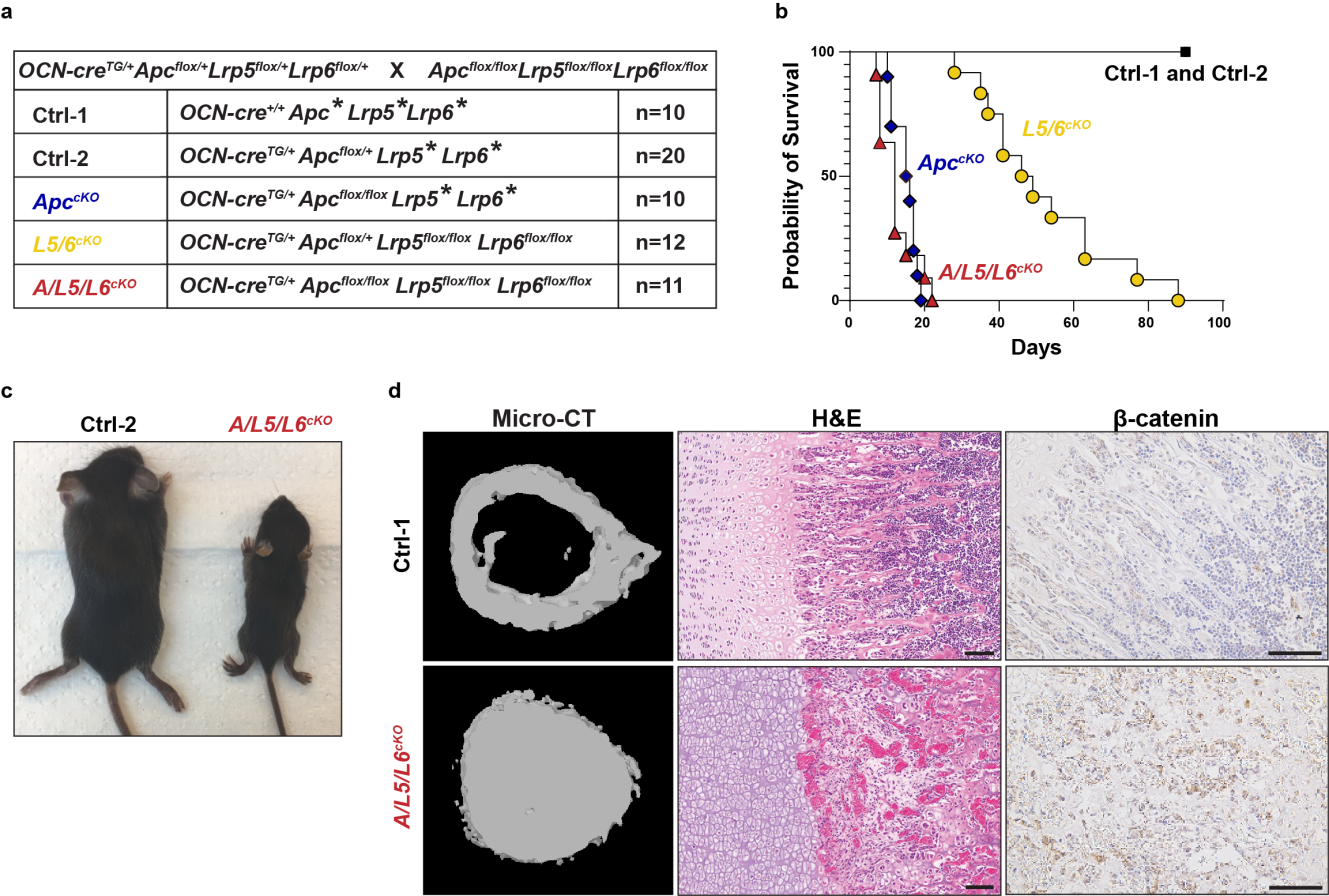
Generation of Apc/Lrp5/Lrp6cKO animals. A) Table indicating the breeding strategy to generate A/L5/L6cKO animals and line abbreviations. An * in the line name denotes either flox/+ or flox/flox. For both the Ctrl-2 and ApccKO an * would not be flox/flox for more than one gene. B) Animal were monitored for 90 days and data are plotted as a proportion of mice in each genotype remaining alive as a function of days of age. C) Appearance of female Ctrl-2 and A/L5/L6cKO littermates at 15 days old. D) Representative micro-CT images of cortical bone in the midshaft of the femur, hematoxylin, and eosin (H&E) staining, and immunohistochemical staining for β-catenin in decalcified femurs from Ctrl-1 and A/L5/L6cKO animals. The scale bar on the images represents 100 µm.

## Description

Wnt/β-catenin signaling is critical for normal development and homeostasis (Zhong et al., 2014) . Signaling is initiated when a Wnt ligand binds to a frizzled receptor and co-receptor LRP5 or LRP6, increasing stabilized, active β-catenin which drives the expression of downstream transcriptional targets. In the absence of Wnt, the adenomatous polyposis coli (APC) protein is required for β-catenin degradation. APC is part of the multiprotein destruction complex which binds β-catenin to facilitate its phosphorylation by glycogen synthase kinase 3 (GSK3) and casein kinase 1 (CK1), targeting it for ubiquitin-dependent proteolysis (Stamos & Weis, 2013) . APC is inactivated in ~80% of all colon cancers (Kwong & Dove, 2009) , and loss of this protein is primarily associated with β-catenin activation because of its requirement in the destruction complex. However, the role of APC may be more complex. *In vitro* data suggests that in the absence of APC, β-catenin stabilization can depend on forming signalosome complexes, which require the presence of co-receptors LRP5 and/or LRP6 (Cabel et al., 2019; Saito-Diaz et al., 2018) . This model has been the subject of debate as groups have conflicting reports on the requirement of LRP5/6 for β-catenin activation after the loss of APC *in vitro* (Cabel et al., 2019; Chen & He, 2019) . We aimed to understand whether LRP5 or LRP6 proteins are necessary for β-catenin stabilization in the absence of APC in an *in vivo* model.

Because global deletion of *Apc* alone (Oshima et al., 1995) or global double knockout of *Lrp5* and *Lrp6* (Kelly et al., 2004) results in embryonic lethality, we used a conditional knockout model. We have extensive published data on how the loss of *Apc* or *Lrp5* and *Lrp6* in osteoblasts impacts bone biology (Craig et al., 2023; Cui et al., 2011; Holmen et al., 2005; Joeng et al., 2011; Riddle et al., 2013) , so we used this same model for our question. Animals with an osteoblast-specific deletion of *Apc* ( *OCN-cre ^TG/+^ ;Apc ^flox/flox^* ) have significantly increased bone mass and bone deposition associated with early perinatal death by 14 days of age (Holmen et al., 2005) . Animals with deletions of both *Lrp5* and *Lrp6* in osteoblasts ( *OCN-cre ^TG/+^ ;Lrp5 ^flox/flox/+^ ; Lrp6 ^flox/flox^* ) develop low bone mass associated with reduced survival (approximately 50% of mice die before seven weeks of age) (Riddle et al., 2013) . Similarly, animals with an osteoblast-specific deletion of *β-catenin* ( *OCN-cre ^TG/+^ ;β-catenin ^flox/flox^* ) have extremely low bone mass and die within four weeks of birth (Holmen et al., 2005) . In addition, mice with simultaneous osteoblast-specific deletion of both Apc and *β-catenin* ( *OCN-cre ^TG/+^ ;Apc ^flox/flox^ ;β-catenin ^flox/flox^* ) phenocopy those carrying only the *β-catenin* deletion, demonstrating that the increased bone mass seen after Apc loss is β-catenin dependent (Holmen et al., 2005) *.*

We reasoned that if the phenotypes caused by loss of APC depend on the presence of LRP5/6, triple knockout mice for *Apc/Lrp5/Lrp6* would mimic the Lrp5/6 knockouts and develop low bone mass. In contrast, if triple knockout mice develop high bone mass, it would indicate that bone phenotypes associated with loss of APC are independent of LRP5/6.

We generated osteoblast conditional knockouts by crossing *Osteocalcin* ( *OCN* ) cre transgenic mice to those carrying floxed *Apc* , *Lrp5* , and *Lrp6* alleles. We used the mating scheme detailed in **Fig. 1a** to generate animals lacking all three genes in osteoblasts, which we will refer to as *A/L5/L6 ^cKO^* . We also generated *OCN-cre* positive mice homozygous for the floxed allele of *Apc* ( *Apc ^cKO^* ) that retained one wild-type *Lrp* allele. Because our previous work showed that retention of at least one allele of either *Lrp5* or *Lrp6* was sufficient to maintain enough bone to support a normal lifespan (Riddle et al., 2013) , we used these mice to evaluate the effects of APC loss in the presence of LRP5/6. We further generated mice in which *OCN-cre* homozygously deletes both *Lrp5* and *Lrp6* but which retain one copy of *Apc* ( *L5/6 ^cKO^* ). Our previous analysis indicated that retention of one functional copy of *Apc* was sufficient to prevent early death and dramatically increased bone mass seen in *OCN-cre;Apc ^Fl/Fl^* mice (Holmen et al., 2005) , so we used these mice to assess the effects of simultaneous loss of *Lrp5/6* .

We monitored the mice from these crosses and noted that some were severely runted and died before three weeks of age ( **Fig. 1b, c** ). Genotyping revealed that these mice were exclusively from either the *Apc ^cKO^* or *A/L5/L6 ^cKO^* cohorts. All *Apc ^cKO^* (n=10) and *A/L5/L6 ^cKO^* knockout (n=11) animals became moribund before 25 days of age, necessitating euthanasia. This is similar to our previous report in which 90% of the *OCN-cre;Apc ^Fl/Fl^* mice died by 3 weeks (Holmen et al., 2005) . We also noted *L5/L6 ^cKO^* knockout mice started dying at 4-5 weeks of age and approximately 50% of our *L5/L6 ^cKO^* genotypic cohort became moribund by 7 weeks of age which corroborated our previous findings with this model. Interestingly, the *Apc* allele in the *L5/L6 ^cKO^* knockout model was heterozygous which suggests that heterozygosity for an *Apc* deletion did not provide a survival advantage for *OCN-cre;Lrp5 ^Fl/Fl^ ;Lrp6 ^Fl/Fl^* mice when compared to our previous work.

Femurs were collected, and micro-computed tomography (Micro-CT) was performed to generate 3D models (Foxa et al., 2021) . Micro-CT allowed us to see bone mineralization differences between the different genotypes. Bones from *A/L5/L6 ^cKO^* animals had dramatically increased mineralized bone in the marrow cavity ( **Fig. 1d** ). These findings mirror those of the *Apc ^cKO^* from this and our previous publication (Holmen et al., 2005) . Histological analysis was performed to gain further insight into the cellular phenotypes supporting the increased bone deposition. Hematoxylin and eosin (H&E) stained sections show increased trabecular bone at the ridge of the growth plate and decreased hematopoietic cells within the bone marrow of *A/L5/L6 ^cKO^* animals. We used immunohistochemistry to look at changes in β-catenin expression within the bone. Looking again at the trabecular bone near the growth plate, we saw an increase in β-catenin expression in cells within *A/L5/L6 ^cKO^* bones. These findings demonstrate that in the absence of APC, β-catenin stabilization is not dependent on LRP5 and LRP6 *in vivo* .

This work is the first to directly evaluate the requirement for LRP5/6 for the increased β-catenin protein levels seen after loss of APC in an *in vivo* model. Our analysis shows that in osteoblasts, stabilization of β-catenin associated with *Apc* inactivation occurs in an LRP5/6-independent manner. It is important to note that the *Apc-flox* allele used in our studies results in the deletion of exon 14 following exposure to cre (Shibata et al., 1997) . Thus, it remains possible that alterations in other locations within the *Apc* locus could cause different effects. In addition, we want to emphasize that tissue-specific differences in the roles of LRP5/6 following the loss of APC may exist. For example, differential expression of other components that regulate β-catenin signaling could influence signaling outcomes in other tissues.

## Methods

**Animals**

Mice with the *Apc (Shibata et al., 1997)* , *Lrp5* , and *Lrp6* (Riddle et al., 2013) conditional knock-out alleles were previously described. Animals used in this study were crossed to an osteoblast-specific Cre ( *OCN-Cre* (Zhang et al., 2002) or also called BGLAP-cre) transgenic mouse to generate a bone-specific *Apc* / *Lrp5* / *Lrp6* triple knockout mouse model. These mice were maintained following institutional animal care and use guidelines, and experimental protocols were approved by the Institutional Animal Care and Use Committee of the Van Andel Institute. Mice were housed in Thoren Maxi-Miser IVC caging systems with a 12-h/12-h light/dark cycle and fed a breeder rodent diet containing 23% protein and 24% fat with an energy content of 19.3 MJ/kg (5021, LabDiet St. Louis MO) with food and water provided *ad libitum* .

For micro-CT (Foxa et al., 2021) and histological analysis, animals were euthanized at 7 days old. Femurs were isolated and fixed in 10% neutral-buffered formalin (NBF) at room temperature for 48 h, then changed to 70% ethanol before Micro-CT imaging and histological processing.

**Genotyping Analysis**

Genomic DNA was isolated from ear punches using alkaline extraction (Truett et al., 2000) . A previously published PCR-based technique was used to genotype the animals (Holmen et al., 2005; Riddle et al., 2013) . Please see the table for genotyping primers.

**Micro-computed tomography (µCT)**

Femurs were analyzed and 3D-modeled using a SkyScan 1172 µCT system (Bruker Micro-CT: Kontich, Belgium). Femora were scanned in 70% ethanol using an X-ray voltage of 50 kV, current of 200 µA, and 0.5 mm aluminum filter with a voxel size of 7.99 µm. Femoral images were reconstructed using NRecon 1.7.4.6 (Bruker Micro-CT). The mineralized tissue was oriented, and a volume of interest (VOI) was defined using DataViewer 1.5.6.3 (Bruker Micro-CT). Regions of interest (ROI) were defined using CTAn 1.18.8.0 (Bruker Micro-CT), and models were generated using CTvol 2.3.2.0 (Bruker Micro-CT).

**Histology and Immunohistochemistry**

Fixed femurs were decalcified in 10% EDTA for 14 d, embedded in paraffin, and 5 um sagittal sections taken from the midshaft were adhered to glass slides. Sections were stained with hematoxylin and eosin (H&E), or immunohistochemistry was performed for β-catenin (Cell Signaling, 9562) expression.

## Reagents

**Table:** 

**Strain**	**Genotype**	**Available From**
** Apc ^tm1Tno^**	Homozygous floxed	VAI (MGI: 1857966)
** B6;129- *Lrp5 ^tm1.1Vari^* /J **	Homozygous floxed	Jackson Labs (026269)
** B6;129S- *Lrp6 ^tm1.1Vari^* /J **	Homozygous floxed	Jackson Labs (026267)
**B6.FVB-Tg(BGLAP-cre)1Clem/J**	Hemizygous	Jackson Labs (019509)

**Table:** 

**Antibody**	**Animal and Clonality**	**Description**
**β-Catenin Antibody**	Rabbit polyclonal	Rabbits were immunized with a synthetic peptide corresponding to residues around Ser37 of human β-catenin. Available at Cell Signaling Technology (#9562).

**Table:** 

**Name**	**Sequence (5’ – 3’)**	**Reference**
**APC-flox-P3**	GTTCTGTATCATGGAAAGATAGGTGGTC	PMID: 9311916
**APC-flox-P4**	CACTCAAAACGCTTTTGAGGGTTGATTC	PMID: 9311916
**Lrp5 Flox-S2**	CCACCAATCATCAGCCAAGGA	PMID: 21602802
**Lrp5 wt-AS2**	TCACCTGTCCTAGTGCAGAAGGA	PMID: 21602802
**Lrp6-Flox-F**	GGGGTTCTACTTTTGTGTGTGG	PMID: 21866564
**Lrp6-Flox-R**	CCGTCTGTTTGCATAAAGCAACA	PMID: 21866564
